# 

**Published:** 1931

**Authors:** 


### CONTENTS.

| | Page |
|---|---|
| **A Review of Seven Hundred Cases of Acute Appendicitis.** By Hubert Chitty, M.S., F.R.C.S. | 167 |
| **The Work of the University Centre of Cardiac Research, 1927-31.** By Carey F. Coombs, M.D., F.R.C.P. | 179 |
| **Sterilization of the Unfit.** By H. C. Bristowe, M.D. | 189 |
| **Tetany following Thyroid Operations.** By Duncan Wood, F.R.C.S. | 205 |
| **A Case of Congenital Obstruction of the Bile-Duct.** By J. A. Birrell, M.D., M.R.C.P., and A. Wilfrid Adams, M.S., F.R.C.S. | 215 |
| **Reviews of Books** | 219 |
| **Editorial Notes** | 223 |
| **Obituary:** Francis Richardson Cross, M.B., F.R.C.S., LL.D., J.P. | 226 |
| **The Medical Library of the University of Bristol** | 229 |
| **Local Medical Notes** | 231 |

